# Integrating Transcriptomics and Metabolomics to Unravel the Molecular Mechanisms of Meat Quality: A Systematic Review

**DOI:** 10.3390/foods15081271

**Published:** 2026-04-08

**Authors:** Kaiyue Wang, Ren Mu, Yongming Zhang, Xingdong Wang

**Affiliations:** 1 College of Life Science and Technology, Inner Mongolia Normal University, Hohhot 010022, China; 17702902641@163.com (K.W.); murensk@imnu.edu.cn (R.M.); zym78@163.com (Y.Z.); 2 Key Laboratory of Biodiversity Conservation and Sustainable Utilization in Mongolian Plateau for College and University of Inner Mongolia Autonomous Region, Hohhot 010022, China

**Keywords:** transcriptome, metabolome, multi-omics integration, meat quality, regulatory mechanism biomarkers, postmortem muscle metabolism

## Abstract

Meat quality serves as a pivotal determinant of consumer purchasing behavior and of the economic viability of the livestock industry; as such, research into its regulatory mechanisms is of critical significance for the development of modern agriculture. Traditional investigations into meat quality have predominantly centered on sensory and physicochemical assessments of ultimate phenotypic traits, thereby facing inherent limitations in systematically deciphering the intricate molecular regulatory networks underlying meat quality formation. By contrast, an integrated analysis of the transcriptome and metabolome effectively connects the cascade of “gene transcription—metabolic regulation—phenotypic determination,” which has emerged as a core methodological paradigm in contemporary research on the molecular mechanisms governing meat quality. This review systematically delineates the evolutionary trajectory and principal technological frameworks of meat quality evaluation systems, with a focused synthesis of recent advances achieved through combined transcriptomic and metabolomic analyses in the field of meat quality regulation. The scope of this review encompasses core transcriptional regulatory networks associated with meat quality attributes, pivotal metabolic pathways, signal transduction mechanisms, and protein degradation dynamics. Furthermore, the regulatory impacts exerted by genetic variation among breeds, nutritional modulation, rearing environments, and stress responses on meat quality characteristics are comprehensively elucidated. Integrative analysis reveals that combined transcriptome–metabolome approaches transcend the inherent limitations of single-omics investigations, systematically unraveling the hierarchical regulatory mechanisms governing fundamental meat quality traits, such as muscle fiber type differentiation, postmortem glycolytic progression, intramuscular fat deposition, and flavor compound accumulation. Such integrative strategies have facilitated the identification of functional genes and metabolic biomarkers with potential utility for the early prediction of meat quality outcomes. Concurrently, this review acknowledges persistent challenges confronting the field, including the absence of standardized protocols for multi-omics data integration, insufficient functional causal validation, and a discernible disconnect between research discoveries and practical industrial implementation. Building upon this comprehensive assessment, prospective directions for future multi-omics research in meat quality are proposed, accompanied by the formulation of an integrated end-to-end improvement framework spanning fundamental research, technological innovation, and industrial application. Collectively, this review provides a systematic theoretical foundation for the in-depth elucidation of mechanisms that determine meat quality and the precision-oriented regulation of quality-determining traits in livestock production practices, thereby offering substantial scientific guidance for quality improvement initiatives within the animal husbandry sector.

## 1. Introduction

The value of livestock and poultry products depends largely on the quality of meat. The study of this trait is not only for the economic benefit of animal husbandry, but it is also closely related to food safety [1,2]. The study of meat quality has undergone nearly a century of development. Prior to the mid-20th century, it mainly relied on empirical sensory evaluation, which centered on subjective consumer preferences and experiential grading in slaughterhouses, lacking a standardized evaluation system and only enabling a rough assessment of meat appearance and palatability. From the mid-20th century to the early 21st century, a standardized objective evaluation system was established. With the systematic development of meat science, physicochemical testing indicators centered on pH value, Warner–Bratzler shear force (WBSF), water-holding capacity, and intramuscular fat content were progressively developed, and organizations such as the American Meat Science Association (AMSA) and the International Organization for Standardization (ISO) successively published a series of testing standards, achieving a leap from subjective description to the objective quantification of meat quality. However, this stage only focused on the detection of ultimate phenotypes and could not elucidate the intrinsic biological mechanisms underlying meat quality formation [3]. From the early 21st century to 2010, with the development of molecular biology techniques, research gradually delved into the level of genes and pathways, identifying a group of core candidate factors regulating meat quality, such as the calpain system and genes related to muscle fiber development. However, limited by technological throughput, research was restricted to the analysis of single genes and individual pathways, and a complete regulatory network could not be constructed. In the current era of multi-omics integration research, breakthrough progress has been made in high-throughput sequencing and mass spectrometry technologies represented by transcriptomics, metabolomics, and proteomics, bringing revolutionary changes to meat quality research. In particular, the combined analysis of the transcriptome and metabolome has broken the limitations of single-omics research, opening up a new avenue for systematically deciphering the complete “gene–metabolism–phenotype” molecular chain mechanism underlying meat quality trait formation [4]. Although this field has gradually evolved from single-indicator evaluation to multi-dimensional comprehensive analysis, it still faces numerous challenges: the lack of a unified system for sensory evaluation (tenderness, juiciness, and flavor scoring), non-standardized detection protocols and threshold settings for instrumental detection indicators (pH value, water-holding capacity, and shear force), as well as notable deficiencies in the depth of molecular mechanism elucidation [5]. The integration of multi-omics data confronts multiple technical bottlenecks, including strong data heterogeneity across different sequencing/mass spectrometry platforms, a lack of unified analysis processes, and incomplete metabolite annotation databases specific to livestock and poultry, which prevents the precise identification of a large number of characteristic metabolites related to meat flavor and tenderness. Furthermore, there is a serious disconnect in the industrial translation application of research results; most of the molecular markers and regulatory targets identified at the laboratory level have not undergone field validation in large populations and diverse scenarios, making it difficult to directly guide practices in industrial sectors such as livestock breeding, nutritional regulation, and slaughtering and processing.

This review systematically summarizes the entire progression of meat quality research, from traditional phenotypic evaluation to multi-omics integrated analysis. It comprehensively synthesizes the core research advances in sensory evaluation, physicochemical detection, molecular marker mining, and the application of multi-omics technologies. Furthermore, it critically examines the core limitations of existing research and the key issues in multi-omics integration, with a particular focus on integrating research findings from transcriptomics and metabolomics. By constructing a complete “gene–metabolism–phenotype” regulatory network underlying meat quality formation, while also clarifying the research potential of cutting-edge directions such as three-dimensional chromatin conformation regulation, this review proposes verifiable research pathways and methodological norms. This review aims to address critical gaps in the existing research, namely the lack of systematic mechanism elucidation, non-standardized data integration, and the disconnect in industrial translation, thereby providing a robust theoretical foundation and technical support for the establishment of a globally unified multi-dimensional meat quality evaluation system, the realization of precision breeding, and the targeted regulation of meat quality.

## 2. Meat Quality Evaluation Indicators and Detection Methods

The scientific evaluation system of meat quality needs to integrate multi-dimensional parameters such as sensory characteristics, physical and chemical indicators and molecular characteristics [6]. Consumers’ purchase decisions are initially influenced by product appearance, while texture characteristics directly determine the sensory experience upon consumption. At the molecular level, the chemical composition of meat is closely related to its nutritional value. In particular, key biochemical indicators such as protein phosphorylation status [7], pH [8], intramuscular fat content [9], ATP metabolism, glycolytic potential, and the overall condition of muscle proteins (which is associated with meat color, water-holding capacity, proteolysis, and oxidation levels) [9] play an important regulatory role in meat quality. With the development of technology, meat quality detection has expanded from traditional sensory evaluation and physical and chemical analysis to the integration of high-throughput technologies such as transcriptomics and metabolomics, and has led to the development of a multi-scale, multi-dimensional modern evaluation system [4,10].

### 2.1. Sensory Evaluation Methods

The sensory evaluation of meat quality is a key link to assess consumer acceptance, and its scientificity and systematicness directly affect the reliability of the research results. Sensory analysis is mainly divided into analytical methods and emotional methods [11]. Quantitative description analysis was adopted for professional evaluation, with the appearance, texture and flavor of meat samples assessed according to the nine-point hedonic scale system [12]. The consumer test focuses on the overall acceptance. The paired comparison method or ranking method is commonly used to collect preference data, and Friedman variance analysis is recommended to process the ranking results [13].

However, the standardization of sensory evaluation faces challenges. The difference in sensitivity of evaluators may lead to data variation, especially in flavor evaluation, as the individual’s olfactory threshold for specific flavor substances is significantly different. In addition, cooking methods (such as final temperature and standing time) can affect protein denaturation and flavor release, so heat treatment parameters need to be strictly controlled and recorded. Although bionic sensors such as electronic tongues and electronic noses can assist in reducing human error, their ability to analyze complex flavor profiles is still inferior to that of well-trained sensory groups.

### 2.2. Analysis of Physicochemical Indicators

The physical and chemical detection indexes of meat quality provide a scientific basis for the objective evaluation of meat quality. Among them, pH value, moisture content and shear force are the key parameters for evaluating meat quality. pH value is an important index to reflect the metabolic status of muscle after slaughter. During the storage of Qinchuan beef, the pH value decreased first and then increased, reaching the lowest value of 5.37 ± 0.03 on the fourth day [14], a trend that was consistent with the decrease in ATP, AMP and NADH content. The change in pH value directly affects the degree of protein denaturation and water-holding capacity, and then affects the tenderness and juiciness of meat. Using ^31^P nuclear magnetic resonance (NMR), a transient postmortem pH increase was observed in sheep muscle, where the pH increased briefly at the early stage after slaughter, and then continued to decrease to the limit value of 5.60–6.07 [15]. The difference may be related to muscle type and individual metabolic characteristics.

Water content is closely related to meat juiciness and economic value. The filter paper press method is commonly used to determine water-holding capacity [16]. Post-slaughter handling methods significantly impact water content; for instance, mechanically deboned poultry meat, due to the omission of washing and cooling steps, suffers from insufficient water absorption, leading to reduced yield. Water loss is correlated with changes in pH value. When the pH approaches the isoelectric point of proteins (5.2–5.4), water-holding capacity is at its weakest, and drip loss increases. The myofibrillar fragmentation index (MFI) is negatively correlated with water-holding capacity. This is primarily because muscle glycolysis causes a rapid pH decline, and sarcomere shortening compresses the space for water retention, significantly reducing water-holding capacity. During this stage, calpains are only initially activated, and the MFI increases slowly; a higher MFI indicates more severe disruption of the myofibrillar structure [14].

Shear force is an objective method to evaluate meat tenderness. WBSF and Slice Shear Force (SSF) are commonly used for measurement [17]. The WBSF test requires that the meat sample be heated to 71 °C and then sampled. The higher the initial temperature of the meat sample, the lower the shear force value. Studies have shown that steaks with WBSF values less than 4.6 kgf are rated as “tender” by more than 88.6% of consumers, and steaks with WBSF values higher than 5.7 kgf are all rated as “unacceptable” [18,19]. However, there are individual differences in consumers’ acceptance of tenderness, and the corresponding relationship between WBSF value and tenderness of beef in different parts also varies. For example, the WBSF value of beef tenderloin is usually low, while the WBSF value of beef chuck roll is relatively high [20]. As an improved method of WBSF, the correlation between SSF and sensory score (r = −0.82) was slightly higher than that of WBSF (r = −0.77) [21].

There are complex interactions between the above indicators. Table 1 summarizes the changes in key quality indicators of post-mortem beef during maturation. The rate of pH decline affects protein denaturation, which in turn affects water-holding capacity and shear force. MFI was significantly negatively correlated with shear force and was associated with the expression of cathepsin D (CTSD), PSMD13 and other proteins. In terms of energy metabolism, ATP depletion and lactic acid accumulation lead to a decrease in pH, while AMP deaminase activation promotes IMP production, accompanied by phosphate release, further regulating muscle tenderization. The dynamic changes in these indexes reflect the biochemical mechanism of the conversion of muscle to edible meat, which provides an objective basis for scientific evaluation and improvement of meat quality.

### 2.3. The Impact of Omics on Meat Quality

#### 2.3.1. Regulation of Post-Disaster Muscle Glycolysis and pH Homeostasis

The postmortem glycolysis process directly determines the trajectory of pH decline and is a core link affecting meat water-holding capacity, color, and the occurrence of meat quality abnormalities. Traditional methods can only reflect the ultimate outcome of glycolysis through pH measurement, and cannot resolve the intrinsic mechanisms underlying the differences in glycolysis rates among different breeds and individuals.

Transcriptomics technology, through sequencing muscle tissue at different postmortem time points, has precisely identified functional gene modules that regulate the activity of key glycolytic enzymes. For instance, genes such as PPP1R11 and PVALB regulate the expression of key glycolytic enzymes via calcium ion signaling pathways, thereby directly influencing the rate of postmortem pH decline and the ultimate pH value [16]. Among these, PVALB, acting as a calcium-buffering protein, can affect the activity of glycolytic enzymes by modulating the concentration of calcium ions in the sarcoplasm, making it a key transcriptional marker for predicting postmortem pH changes [23].

Metabolomics techniques have further filled the gap in detecting the dynamic changes in glycolytic intermediates. Through non-targeted metabolomic analysis, the dynamic trajectories of metabolites such as ATP, AMP, lactate, and glycogen have been elucidated. A predictive model that links the content of glycolytic intermediates within 2 h postmortem to the ultimate pH value and water-holding capacity has been established, enabling early warning of postmortem meat quality abnormalities such as PSE (pale, soft, and exudative) meat and DFD (dark, firm, and dry) meat. This overcomes the lag of traditional methods, which require waiting 24 h postmortem to determine abnormal meat quality [24,25].

#### 2.3.2. Regulation of Myofibrillar Degradation and Tenderization Process

Tenderness is a core textural attribute determining the economic value of meat. With the advancement of phosphorylated proteomics technologies, the regulatory mechanisms by which reversible phosphorylation modifications of myofibrillar proteins and the calpain system govern the tenderization process have been systematically elucidated: the phosphorylation levels of myosin heavy chain and actin directly influence their degradation rate, while the phosphorylation modification of calpastatin can regulate the activity of calpains, acting as a core molecular switch in the postmortem tenderization process [26]. This discovery fills the gap left by traditional research, which could only detect calpain expression levels but could not resolve the regulation of their post-translational modifications, thereby providing a direct target for the precise regulation of tenderness.

The integrated analysis of transcriptomics and metabolomics has further constructed a molecular network for tenderness regulation. In studies on livestock such as pigs and cattle, functional genes closely related to tenderness, including *SFRP2*, *CIB2*, and *UCHL1*, have been identified. Furthermore, it was discovered that the content of nucleotide metabolites such as ATP, ADP, inosine, and hypoxanthine was significantly elevated in the muscles of the high-tenderness group. This establishes an association model linking “gene expression—metabolite changes—tenderness phenotype” [27,28]. Predictive models based on these molecular markers can achieve tenderness grading in the early postmortem period, with an accuracy rate exceeding 90%, thereby overcoming the limitations of traditional shear force detection, which requires heat treatment and destruction of the samples.

#### 2.3.3. Regulation of Lipid Metabolism and Flavor Quality

Flavor is the core sensory attribute determining consumer acceptance. Gas Chromatography–Mass Spectrometry (GC-MS) targeted metabolomics technology has overcome the subjective limitations of traditional flavor evaluation and systematically resolved the composition and origin of volatile flavor compounds in meat. In research on the regulation of mutton off-flavor, by comparing the metabolic profiles of mutton with different intensities of “off-flavor,” branched-chain fatty acids and their precursors were successfully identified as key differential metabolites, subsequently enabling the reduction in off-flavor [29]. In their study on spoiled dry-cured ham, Liao Renyong et al. [30] used Principal Component Analysis (PCA) and Cluster Analysis (CA) to determine that oligopeptides and amino acid derivatives were important metabolic markers distinguishing normal ham from spoiled ham. Combined with PLS-DA model analysis and metabolic pathway analysis, they further confirmed that purine metabolism, pyrimidine metabolism, and protein degradation are the main metabolic pathways affecting the spoilage of dry-cured ham.

Liquid Chromatography–Tandem Mass Spectrometry (LC-MS) non-targeted metabolomics technology has further revealed the metabolic pathways of flavor precursors. Through a comparative analysis of Laiwu pigs and Yorkshire pigs, it was determined that amino acid metabolites such as oxoglutarate and L-aspartate are core substances of meat flavor precursors, constructing a regulatory network of “lipid metabolism—amino acid metabolism—volatile flavor compounds” and providing a theoretical basis for the precise regulation of flavor quality [31]. Simultaneously, metabolomics technology enables early monitoring of flavor deterioration during meat storage by detecting dynamic changes in lipid oxidation metabolites, overcoming the limitation of traditional methods that can only reflect the terminal oxidation degree through indicators such as peroxide value and acid value [32,33].

#### 2.3.4. Microbial Metabolism and Regulation of Storage Stability

Microbial proliferation during meat storage is a primary factor leading to quality deterioration. Traditional methods can only reflect the final contamination status through total viable counts, but fail to elucidate the interaction mechanisms between microbial communities and meat quality deterioration, consequently resulting in insufficient early warning capability.

Metatranscriptomics technology, by analyzing the gene expression profiles of microbial communities during storage, has identified the core microbial populations responsible for meat spoilage and their associated metabolic pathways. It has revealed the interaction mechanisms between microbial metabolism and muscle protein degradation as well as lipid oxidation, thereby providing a theoretical basis for the targeted development of meat preservatives and the optimization of storage processes [34]. Metabolomics technology, by detecting changes in microbial metabolic markers within the meat, enables early warning of quality deterioration before the total viable count exceeds permissible limits, significantly enhancing the capability for quality monitoring during meat storage.

### 2.4. Development of Molecular Markers

One of the core application values of omics technologies lies in discovering molecular markers that can be used for the early prediction of meat quality and for breed selection, thereby overcoming the lag inherent in traditional evaluation methods. To date, research based on transcriptomics, metabolomics, and proteomics has identified a large number of molecular markers closely associated with meat quality traits. In the context of breed selection, studies on Qinchuan cattle have confirmed that selection strategies based on single-nucleotide polymorphism (SNP) markers can effectively improve core meat quality traits such as intramuscular fat content and tenderness [35]. Such markers can directly reflect the physiological state of muscle tissue and the progress of meat quality formation; compared to genomic markers, they are closer to the ultimate phenotype and offer significant advantages in the precise grading of meat quality. In research on Silkies chickens, 13 characteristic metabolic markers, including estradiol, niacin, and creatine, have been identified and can be used for quality grading of chicken meat with high nutritional value [36].

## 3. Molecular Mechanisms Regulating Meat Quality

The formation of meat quality involves a multi-level molecular regulatory network, which is composed of gene expression regulation, metabolic pathway activation and signal transduction systems [37]. Recent studies have found that post-translational modifications, such as protein phosphorylation, have important regulatory functions in the post-slaughter muscle transformation stage. These modifications directly affect the conformational changes in myofibrils and the degradation kinetics of myofibrillar proteins [38,39]. The signal transduction system plays an important role in regulating the growth, differentiation and metabolism of muscle cells, and the MSTN/Smad signaling pathway is one of the important pathways regulating muscle growth [40]. The in-depth study of these regulatory mechanisms will help to fully understand the biological nature of meat quality formation, and provide theoretical bases and technical means for the precise regulation of meat quality [41].

### 3.1. Transcriptional Regulatory Networks

The transcriptional regulatory network is a key regulatory system for meat quality formation, and its mechanism depends on the complex interaction network between transcription factors and target genes [42]. In the study of fat deposition and muscle traits in pigs, the expression characteristics of zinc finger transcription factor *ZNF24* are significantly correlated with specific alleles (such as Duroc alleles), which provides an important basis for revealing the genetic mechanism of meat quality traits [43]. It is worth noting that the complexity of transcriptional regulation not only stems from the functional diversity of a single transcription factor, but also is reflected in the synergy of multi-level regulatory networks [44]. Studies of Arabidopsis flower development have identified 568 feed-forward loop (FFL) patterns that demonstrate how master transcription factors construct sophisticated cascade regulatory networks by simultaneously regulating miRNAs and their targeted transcription factors [45]. Although the research objects are plants, the regulatory principles it reveals offer important insights into understanding the transcriptional network mechanism of animal muscle development.

With the rapid development of transcriptomics technology, new research tools have been obtained to analyze the transcriptional regulatory network of meat quality traits. The application of Ribonucleic Acid Sequencing (RNA-seq) and Single-Molecule Sequencing (SMS) technology has enabled researchers to comprehensively quantify gene expression levels, discover new transcripts, and identify alternatively spliced genes [46]. In the study of pig muscle tissue, the effect of different fiber type ratios on gene expression in pig longissimus dorsi muscle was analyzed by transcriptional composition, and genes related to slow muscle fiber (type I) and fast muscle fiber (type IIB) were identified [47]. Damon et al. [48] compared the muscle transcriptomes of Large White and Basque pigs with different meat quality phenotypes, and found genes related to muscle metabolism and structure. Leal-Gutiérrez et al. [49] identified the cytoskeleton structure genes and pathways related to beef tenderness by RNA-seq analysis. These results provide important data support for the construction of a complete transcriptional regulatory network of meat quality traits.

The combined analysis of metabolomics and transcriptomics has opened up a new way to understand the functional mechanism of transcriptional regulatory networks. As the end product of gene transcription and protein modification, the composition of metabolites directly reflects the physiological state of cells [50]. In the study of meat quality, the causal relationship between transcriptional regulation and metabolic phenotype can be effectively revealed by the association analysis of differentially expressed genes and differential metabolites [51]. The detection, annotation and relative quantification of more than 1000 endogenous and exogenous metabolites by GC-MS and LC-MS techniques provide direct evidence for understanding the metabolic effects of transcriptional regulation [52]. This multi-omics-integrated research strategy has been widely used in meat quality research, providing a basis for the study of the molecular mechanisms behind meat quality traits.

### 3.2. Metabolic Pathway Analysis

As a key bridge connecting gene expression and phenotypic characteristics, metabolic pathways play a decisive role in the formation of meat quality. The glucose metabolism pathway showed significant dynamic changes in the post-slaughter muscle maturation stage, affecting meat tenderness. The contents of ATP, ADP, AMP and NADH in the longissimus dorsi muscle of Qinchuan beef showed the most significant downward trend during 0~2 days of storage [14]. This dramatic change in energy metabolism directly affects the glycolysis process, and then changes meat tenderness by regulating key indicators such as pH value and myofibril fragmentation index. Under hypoxic conditions, muscle energy metabolism mainly depends on the glycolysis pathway and the creatine kinase reaction (Lohmann reaction), in which glycogen acts as the initial substrate to produce three molecules of ATP and two molecules of pyruvate through the glycolysis pathway [53]. AMP deaminase plays a crucial role in regulating muscle energy metabolism, and its encoding gene, AMPD, is significantly upregulated, especially in muscle atrophy [54].

Lipid metabolism pathways showed obvious specificity among different species and varieties. During the development of yellow-feathered broilers, genes such as *ACOT9*, *CETP*, *LPIN1*, *DGAT2*, and *RBP7* are closely related to intramuscular fat (IMF) deposition. Among them, *ACOT9* and *LPIN1* promote lipolysis, whereas *CETP* reduces fat accumulation by participating in cholesterol reverse transport [55]. Liu et al. [56] found that the intramuscular fat content of Chinese local pig breeds, such as Taoyuan black pigs, was negatively correlated with organic acids such as fumaric acid and succinic acid in muscle, while lipid metabolites (such as 2-hydroxyisovaleric acid) were positively correlated compared with modern breeds such as Duroc pigs, revealing the metabolic regulation network of muscle–adipose tissue interaction. Studies on Polish pig breeds have found that the expression of lipid metabolism-related genes in the longissimus dorsi and biceps brachii muscles is increased (e.g., *PPP1R11*). These genes are involved in the regulation of fat deposition and lipid metabolism [16].

Skeletal muscle is the core organ of energy metabolism, and its endocrine function has an important impact on the overall metabolic homeostasis. Muscle factors secreted by skeletal muscle, such as IL-6, IL-15, IGFBP-5 and insulin-like growth factor 1 (IGF-1), can regulate glucose, lipid and protein metabolism [57,58]. Osteocrin mRNA exhibits a tissue-specific expression pattern in mouse skeletal muscle, and its expression level is tightly regulated by dietary nutritional status. This gene (Osteocrin) regulates IMF deposition and muscle tenderness traits by mediating glucose metabolism, adipogenic differentiation and myofiber development in skeletal muscle; it is an important candidate functional gene affecting core meat quality traits, including meat tenderness and marbling grade [59]. During postmortem aging, changes in energy metabolism are closely related to the mechanism of protein degradation. Myosin heavy chain breaks and the decreased expression of myosin light chain 1,2 and heavy chain 1 directly affect the final meat tenderness [60].

### 3.3. Signal Transduction Mechanisms

Cell signal transduction is a key process that connects external stimuli with intracellular responses, and plays an important role in the regulation of meat quality [61]. Hormone signaling pathways play precise regulatory roles in muscle growth and fat deposition, in which IGF-1 promotes protein synthesis and muscle fiber hypertrophy through the PI3K/Akt/mTOR signaling cascade [62]. Li et al. [63] showed that *SIRT5* regulates lipid deposition in goat preadipocytes through the PI3K-Akt and *MAPK* signaling pathways. It is worth noting that this pathway remains active during postmortem muscle maturation, and its ability to regulate glycogen decomposition rate and lactic acid accumulation directly affects the pH value and water-holding capacity of the final meat [64,65]. Leptin, secreted by adipose tissue, regulates energy metabolism through the JAK2/STAT3 signaling pathway and plays an important role in intramuscular fat deposition [66]. The comparative study of Landes geese and Sichuan white geese showed that differences in the expression levels of the leptin receptor gene were closely related to the degree of liver fat deposition, which provided a molecular basis for understanding the difference in poultry meat quality [67].

### 3.4. Protein Degradation Mechanisms

Protein degradation is a key biological process affecting the formation of meat tenderness, and its regulatory mechanism mainly depends on the complex protease system in cells [68]. The degree of degradation of myofibrils and cytoskeletal proteins directly determines the tenderization effect of meat. Studies have confirmed that the calpain system can specifically decompose structural proteins such as titin, nebulin, filamin, desmin and troponin-T, thereby significantly weakening the mechanical strength of muscle fibers, thereby affecting meat tenderness [68,69,70]. As the core enzyme system regulating meat tenderness, calpain is composed of a variety of isozymes, and its activity is strictly regulated by the endogenous inhibitor calpastatin [71]. There is a synergistic effect between calpain and caspase-3 during chicken postmortem aging. When chicken muscle was treated with inhibitors of calpain and caspase-3, the activity and expression of calpain and caspase-3 were changed [72]. The change in calcium ion concentration is the key factor to activating this system, and environmental conditions such as pH value and ionic strength will significantly change the binding characteristics of calpain and calpastatin [73]. The disorder of energy metabolism in muscle after slaughter leads to a decrease in pH value, which leads to an increase in calcium ion concentration, and finally triggers the activation of the calpain system and initiates the degradation cascade of myofibrillar protein.

Lysosomal protease CTSD plays an important role in protein degradation and apoptosis. It was observed that the higher the expression level of *CTSD*, the more myofibrillar protein degradation, and its expression level showed a significant negative correlation with shear force, while it showed a significant positive correlation with MFI [74]. The reduction in lysosomal membrane stability caused by the decrease in pH leads to the release of *CTSD*, which then participates in the process of protein degradation. The upregulation of *CTSD* expression may accelerate the destruction of myofibrillar structure by enhancing the level of autophagy in muscle cells [75,76]. At the same time, the expression of 26S proteasome regulatory subunit *PSMD13* is also closely related to the improvement of meat tenderness. Protein–protein interaction network analysis showed that *PSMD13* was functionally related to a variety of ATP metabolism-related proteins, suggesting that energy metabolism may affect protein degradation efficiency by regulating proteasome activity [14].

During postmortem muscle ripening, the effects of energy metabolism, pH change and MFI on tenderness showed obvious temporal characteristics. Energy and pH value in yak (*Bos grunniens*) muscle changed most significantly in the early stage (0~4 days) post-slaughter, and this change mainly affects tenderness by regulating the contraction state of muscle fibers. In the later period (4~8 days), as the energy metabolism tends to be stable, the synergistic effect of low pH and endogenous enzymes promotes the continuous increase in MFI, showing a significant lag effect [77]. The transcriptional regulation of protein degradation involves the expression changes in multiple genes. In the PUL muscle, the expression levels of genes involved in protein degradation, such as *CST6*, *CTSD*, *ISG15*, and *UCHL1*, were significantly upregulated [16]. These genes may affect the final tenderness of meat by regulating protease activity and protein stability. It is worth noting that the calcium signaling pathway-related proteins CAMK2D and PPP3R1 show a synergistic effect in promoting apoptosis, both of which jointly promote the increase in MFI and the destruction of myofibrillar structure [14], which provides a new research direction for further understanding the molecular mechanism of protein degradation.

## 4. Impact of Environmental Factors on Meat Quality

As key external factors, the rearing environment and nutritional conditions regulate the formation of meat quality through multi-level biological mechanisms. Studies have shown that factors such as floor type, stocking density, and photoperiod in the breeding environment significantly influence the physicochemical properties of duck meat, including fat deposition, muscle fiber composition, and feed efficiency [78]. Nutritional regulation directly determines meat tenderness, flavor, and nutritional value by intervening in core processes such as lipid metabolism and protein turnover [79]. Meanwhile, environmental stressors (such as temperature fluctuations and stocking density pressure) activate cellular signaling networks, inducing adaptive changes in muscle tissue at the metabolomic and transcriptomic levels, ultimately manifesting as differences in product quality [80]. Notably, the pre-slaughter transportation and slaughtering process are critical nodes that induce acute stress in livestock and poultry, leading to meat quality deterioration, and their impact on final meat quality can even exceed that of chronic environmental regulation during the rearing period [81]. The synergistic effect of environmental factors and nutritional strategies provides an important basis for the systematic improvement of meat quality.

### 4.1. Influence of Rearing Conditions

#### 4.1.1. Precise Regulation of Ambient Temperature and Humidity

Environmental temperature and humidity are the key parameters affecting the quality of poultry meat. In a study on white feather ducks, the body weight and feed intake of 42-day-old ducks decreased significantly at 20 °C compared with 26 °C [82]. Studies have found that chronic heat stress can change the expression profile of miRNAs. For example, in poultry breast muscle, chronic heat stress inhibits glycogen synthesis through the gga-miR-212-5p/GYS1 axis, resulting in a decrease in muscle glycogen content and affecting meat quality [83]. In the lungs of meat ducks, the differential expression of miRNAs such as miR-146 and miR-217 may be involved in a complex gene regulatory network, which mediates heat stress response by affecting the expression of their target genes [84]. In addition, studies on broilers also showed that high temperature environment reduced the pH value and water-holding capacity of breast muscle and activated the expression of heat shock proteins (HPSs), such as HSP70, which confirmed the results of duck studies and revealed the common stress response mechanism across species [82].

#### 4.1.2. Floor Type, Stocking Density and Rearing Systems

Different rearing systems directly determine the activity range, sanitary conditions, and stress levels of livestock and poultry, thereby affecting meat quality. Comparative studies have found that Nonghua ducks raised in a net rearing system showed increased fat deposition in August and January, and enhanced serum antioxidant enzyme (GSH-Px) activity during the late reproductive period. However, the litter rearing system demonstrated advantages in improving slaughter yield, breast muscle weight, and reducing drip loss [85]. The main reason is that the net-rearing system significantly limits the activity range of livestock and poultry, reduces daily energy consumption, and redirects surplus energy toward adipose tissue. Transcriptomic studies confirmed that under the net rearing mode, the transcript levels of key fatty acid synthesis genes FAS, ACC, and SCD1 were significantly upregulated in the liver and abdominal adipose tissue of meat ducks, whereas the expression of genes related to the PPARα pathway, which regulates fatty acid catabolism, was significantly downregulated, thereby directly driving increased fat deposition [86]. In contrast, the litter floor rearing mode, which allows livestock and poultry more freedom of movement on the ground, significantly enhances the activation level of muscle satellite cells. Transcriptomic analysis revealed that in the breast muscle of meat ducks raised on litter, the expression of MyoD and MyoG, core regulatory genes for myofiber differentiation, was significantly upregulated, thereby directly promoting myofiber proliferation and differentiation, and improving breast muscle weight and slaughter performance [82]. In terms of feeding density, studies on ducks have confirmed that high-density feeding can be used as a stress source to induce high expression of the *HSP70* gene and to fully activate the MAPK stress signaling pathway. This leads to excessive accumulation of ROS in muscle cells and alters lactate dehydrogenase activity, resulting in a decrease in meat pH and water-holding capacity [87]. Similarly, in broiler breeding, feeding environments above standard density were also observed to increase serum cortisol levels (a stress indicator), resulting in decreased meat quality [88].

In the study of Hezuo pigs, it was found that compared with simple barn feeding, grazing and supplementary feeding could significantly increase the pH_24_h and pH_45_min of the pork. The reason is that the slow-twitch muscle fiber marker gene MYH7 is significantly upregulated, while the fast-twitch muscle fiber marker gene MYH4 is significantly downregulated, leading to a notable increase in the proportion of oxidative muscle fibers. Oxidative muscle fibers have a higher myoglobin content, which directly enhances meat redness. Concurrently, their postmortem glycolysis rate is more gradual, resulting in a more stable pH decline, which effectively prevents rapid protein denaturation, significantly reduces drip loss and cooking loss, and improves the water-holding capacity of the muscle [89]. A study on Gangba sheep showed that although barn feeding significantly increased final body weight, average daily gain, carcass weight, net meat weight, slaughter rate and meat–bone ratio in sheep, barn-reared mutton had higher brightness (L), cooking loss rate and shear force, and lower pH_24_h in terms of meat quality, resulting in poorer processing quality and eating taste than those of grazing mutton [90]. Different rearing systems have their own advantages and disadvantages regarding their impact on livestock and poultry meat quality; for instance, various systems such as cage rearing, free-range rearing, and net rearing also yield different effects on meat quality [91].

#### 4.1.3. Ecological Farming and Nutritional Interventions

Ecological breeding models, such as the rice–duck farming system, have been shown to improve duck meat flavor and texture [92]. Studies indicate that Jinding ducks raised in this system exhibit significantly higher breast muscle color scores, thinner and denser muscle fibers, and a trend toward increased levels of umami amino acids and total amino acids in the breast muscle [92]. Furthermore, research on geese has found that under similar ecological models, the proportion of unsaturated fatty acids in their muscles increases [93]. In terms of nutritional intervention, studies on broilers have found that magnesium supplementation can inhibit the excessive activation of cellular oxidative stress and apoptosis pathways, reducing muscle cell damage and myofibrillar protein denaturation. It can also suppress the excessive release of calcium ions from the sarcoplasmic reticulum, downregulating the gene expression and enzyme activities of key glycolytic enzymes (HK2, LDHA), thereby reversing the disturbances in muscle glycolytic metabolism caused by heat stress. This reduces the excessive accumulation of postmortem lactate, maintains a stable decline in muscle pH, fundamentally improves muscle water-holding capacity, and significantly enhances the meat quality of broilers subjected to dietary and heat stress [94]. In research on weaning-stressed piglets, combined transcriptomic and metabolomic analysis revealed that dietary supplementation with glutamate significantly inhibited the excessive activation of the NF-κB inflammatory pathway in muscle, downregulated the gene expression of pro-inflammatory factors such as TNF-α and IL-6, and reduced the levels of inflammation-related metabolites in both serum and muscle. This provided molecular-level validation of its core mechanism in alleviating immune stress and improving meat quality [95].

#### 4.1.4. Elucidation of Molecular Mechanisms Through Omics Analyses

The application of molecular biology technology has profoundly revealed the internal mechanism of the feeding environment regulating meat quality. For example, transcriptome analysis showed that *PLIN1*, *PNPLA3* and other genes were differentially expressed among the core genes of lipid metabolism in chicken muscle tissues under different feeding methods. Metabolomics data further showed that eicosane and triglyceride components were significantly enriched in lipid metabolism-related processes. Through the combined analysis of transcriptomics and metabolomics, *NAMPT* and *NMNAT2* were proven to be the key regulators of nicotinic acid and nicotinamide metabolism [96]. In addition, some studies have found that under cold stress conditions, lncRNA in animal cells can regulate the metabolism and physiological functions of cells by regulating the expression of target genes. By constructing a miRNA-lncRNA-mRNA regulatory network, the potential role of lncRNA and circRNA in animal response to environmental stress is revealed, which provides a new perspective for understanding the formation of meat quality traits from the epigenetic level [91].

### 4.2. Regulation by Nutritional Factors

The nutritional status of animals will comprehensively determine the final quality of meat by affecting energy metabolism, fat deposition, protein modification and intestinal microorganisms [97,98]. Studies have found that animal nutritional status is closely related to muscle glycogen content, which is a decisive factor affecting postmortem meat quality [99]. Studies on Tibetan sheep have shown that starvation stress can lead to changes in AMPK activity and glycogen content in muscle, which directly affects the final meat quality [100]. Glycogen rNOE imaging (glycoNOE), which has been successfully applied in humans recently, can reveal three typical recovery modes of muscle glycogen after glycogen consumption induced by exercise with high spatiotemporal resolution. This technology is expected to be applied in the field of animal husbandry in the future [101]. The RNA-Seq study of chickens showed that there were significant differences in the expression of genes related to energy production, muscle development, lipid metabolism and immune response in different strains selected according to the final pH value of breast muscle [102]. A study on the longissimus dorsi muscle of sheep also revealed for the first time that lactic acid can not only reduce the pH value, but also act as a signal molecule to regulate the energy metabolic flow of postmortem muscle by modulating the phosphorylation and acetylation of key enzymes such as glycogen phosphorylase (PYGM) [103].

In terms of intramuscular fat deposition, it was found that the IMF content and taste of Hainan black goat were better than those of Nubian black goat, which was closely related to the active expression of 17 key genes in fatty acid biosynthesis, glutamate metabolism and other pathways [104]. Long et al. [105] also compared the meat quality of Guizhou black goat, Nubian black goat and their hybrid offspring and found that the meat quality of hybrid offspring was significantly improved. Studies on pigs have shown that the proportion of slow muscle fibers in local breeds with better meat quality (such as Taoyuan black pig and Xiangcun black pig) is significantly higher than that of commercial pig Duroc, and is related to higher water-holding capacity and IMF content, which reveals that nutrition and genetic background can affect meat quality by regulating muscle fiber type composition [106].

Vitamins play a diverse role in the regulation of meat quality. Vitamin E, with its antioxidant properties, can effectively delay lipid oxidation, thereby significantly improving the stability of meat color and prolonging the shelf life of products [107]. Moderate restriction of vitamin A intake was observed to help improve the marble pattern score of muscle, which may be related to the regulation of vitamin A on adipocyte differentiation. A study of Angus cattle found that limiting vitamin A intake can increase intramuscular fat by 46% [108]. At the same time, a diet rich in vitamin A affects the color of fat, causing it to appear orange or yellow [109].

Recent studies have also revealed the important role of intestinal microorganisms and their metabolites in the formation of meat quality. Studies on pigs have found that the abundance of Lactobacillus in the colon of local breeds is high, which can promote the synthesis of kynurenic acid through the tryptophan metabolic pathway, thereby activating the AMPK/PGC-1α signaling pathway in muscle and upregulating the proportion of slow muscle fibers [106]. The fecal microbiota transplantation (FMT) experiment confirmed that the level of kynurenic acid and the expression level of AMPK in the muscle of mice transplanted with intestinal flora of local breeds of pigs increased, and the proportion of slow muscle fibers showed an upward trend, which directly proved that intestinal microorganisms can regulate meat quality through specific metabolite nutritional signals [106]. These techniques reveal the mechanism of nutritional factors affecting meat quality by regulating complex networks such as glycogen metabolism, fat deposition, protein modification and key signaling pathways. These findings provide a theoretical basis and potential molecular targets for the development of precise nutritional strategies to improve meat quality.

### 4.3. Stress Response Mechanisms

Environmental stress significantly changes meat quality characteristics by regulating gene expression and metabolic pathways, and its mechanism has been deeply studied in a variety of livestock and poultry. In broiler production, environmental stress can induce immune stress, resulting in reduced feed intake and growth inhibition [110]. Zheng et al. [110] found that immune stress can promote the degradation of branched-chain amino acids, reduce the secretion of growth hormone and insulin-like growth factor-1, thereby inhibiting muscle anabolism. In addition, heat stress, as the main environmental stress source of livestock and poultry breeding, can cause animal malaise, reduced feed intake, and lead to a series of physiological and biochemical changes, such as changes in hormone levels, changes in blood protein levels, and decreased antioxidant capacity [111].

The hormonal regulatory network plays a central role in stress responses. Research indicates that stressful conditions alter the hormonal balance in animals, such as by inducing elevated cortisol levels, which subsequently influence meat quality characteristics through various pathways [111]. A study randomly allocating male piglets into a cortisol group and a control group showed that when serum cortisol levels were significantly elevated, significant changes occurred in meat color at 1 h postmortem, muscle bundle ratio, apoptosis rate, and the expression levels of calcium channel and apoptosis-related genes (*SERCA1*, *IP3R1*, *BAX*, *Bcl-2*, and *Caspase-3*) [112]. Utilizing high-throughput sequencing technologies, it has been discovered that in response to heat stress, muscle cells maintain protein homeostasis by upregulating the expression of HSP family members, and changes in the expression of HSP70 and HSP90 are significantly associated with the rate of postmortem muscle protein degradation [113]. Oxidative stress signaling pathways are active during the postmortem aging process of muscle. ROS induce the release of inflammatory factors via the NF-κB signaling pathway, thereby regulating myofibrillar protein degradation, with ROS levels peaking on day 3 [114]. Studies have shown that the interaction between oxidative stress and protein S-nitrosylation positively modulates the tenderization process of yak meat [115]. Cytokine signaling pathways possess multiple functions in muscle growth, development, and metabolic regulation. IL-6 family cytokines secreted by muscle activate the JAK/STAT signaling pathway through the gp130 receptor, participating in muscle regeneration and metabolic regulation [116]. Members of the transforming growth factor-beta (TGF-β) superfamily, such as myostatin, limit excessive muscle growth by inhibiting the Akt/mTOR signaling pathway, and their gene polymorphisms are associated with muscle growth rate and tenderness characteristics in different cattle breeds [117]. Comparative transcriptome analysis between double-muscled cattle breeds and conventional cattle breeds revealed that significant differences in myostatin expression levels affected muscle fiber type composition and collagen content, ultimately leading to the differentiation of meat quality characteristics [118]. The Wnt/β-catenin signaling pathway regulates the activation of muscle satellite cells and the transformation of muscle fiber types; an increased proportion of slow-twitch fibers contributes to improved meat water-holding capacity and flavor compound accumulation [119,120], offering new perspectives for understanding differences in meat quality. Table 2 illustrates the key metabolic pathways influencing meat quality.

Important progress has also been made in the study of nutritional intervention strategies for stress. As feed additives, polysaccharides can promote the development of immune organs, protect intestinal mucosal barrier function, and alleviate oxidative stress by increasing the activity of antioxidant enzymes [121]. In low-birth-weight piglets, weaning stress has a significant effect on them; glutamate supplementation can reduce immune stress indicators during weaning and after lipopolysaccharide challenge, including reducing TNF-α levels and improving stress recovery ability [122]. These findings at the molecular level have laid a theoretical foundation for the development of targeted nutritional intervention and feeding management strategies, which are helpful to alleviate the adverse effects of environmental stress on meat quality.

## 5. Breed Variations and Genetic Regulation

The variety difference and genetic regulation mechanisms of meat quality are the core issues of meat science research. The wide application of multi-omics technology enables us to systematically analyze the genetic nature of meat quality differences between different species and varieties from the genome to transcriptome and metabolomics. Studies have shown that there are significant differences in meat quality traits among different species such as pigs, goats, chickens and rabbits, and there are complex molecular regulatory networks behind them [51,123]. A comparative developmental genetics study between Chinese native pigs (e.g., Tongcheng pigs) and Western commercial pigs (e.g., Landrace pigs) identified *WNT5B* as a key gene regulating skeletal muscle development. Its expression activity exhibits significant variation across different growth stages and is particularly pronounced in slow-twitch muscle fibers, which are known to influence meat quality and tenderness [124]. The comparative study of Hainan black goat and Nubian black goat revealed the mechanism of meat quality difference from three dimensions: muscle fiber characteristics, metabolite composition and gene regulatory network. Seventeen key genes and multiple important metabolites were identified, which were enriched in four core pathways: fatty acid biosynthesis, glutamate metabolism, linoleic acid metabolism and taurine metabolism [104]. In white feather broilers, the *PPP1CC* gene was identified as a key causal gene affecting the brightness and redness of breast muscle color, and its decreased expression would lead to an increase in the incidence of PSE-like meat [125]. The comparative study of Shuxing No.1 meat rabbit and Ira rabbit screened *SMTNL1*, *PM20D*2 and *EDN1* as key genes that may lead to quality differences and found that volatile metabolites such as 2-undecenal and 4-ethyloctanoic acid were positively correlated with meat flavor [126].

With the deepening of research, a series of genetic variations and genes closely related to meat quality traits have been identified and have become a powerful tool for molecular marker-assisted breeding. In Jiangkou Luobo pigs, multiple SNP loci of the *PPP3CA* and *PPP3R1* genes were confirmed to be associated with meat quality [127]. Another study on hybrid black pigs found that the polymorphisms of *MASTR* and *H-FABP* genes were significantly associated with key meat quality traits such as water loss rate, intramuscular fat content and meat color score. The key genes, such as *MEF2C*, *PPARA*, *GPT2*, and metabolites, such as IMP (inosinic acid) and carnosine, screened by research on Hainan black goat can be used as effective markers for the molecular breeding of high-quality meat goats, and help to facilitate the transition from phenotypic to genotype-based selection [104].

Latest research has begun to delve into the three-dimensional structure of chromatin to explore the regulatory mechanisms of meat quality. Among these, Super-Enhancers (SEs), defined as clusters of high-density active enhancers forming transcriptional regulatory hubs, possess transcriptional activation capabilities far exceeding those of typical enhancers. By recruiting substantial amounts of transcription factors and cofactors, SEs precisely regulate key functional genes determining core meat quality traits, such as skeletal muscle development and fat deposition, thereby serving as a core bridge connecting the reprogramming of three-dimensional chromatin conformation with phenotypic differences in meat quality. Recent three-dimensional genomic studies in porcine skeletal muscle have systematically confirmed that the dynamic reprogramming of chromatin’s three-dimensional architecture is an upstream core mechanism regulating the direction of muscle fiber type differentiation. This research, utilizing oxidative soleus muscle and glycolytic extensor digitorum longus muscle from Duroc pigs, employed high-throughput chromatin conformation capture (Hi-C) integrated with multi-omics sequencing technologies such as ChIP-seq and ATAC-seq. This approach generated high-resolution maps of three-dimensional chromatin conformation and cis-regulatory elements in skeletal muscles of different metabolic types, systematically elucidating the regulatory function of super-enhancer-mediated three-dimensional chromatin interactions in myofiber differentiation [128]. Studies have found that in pig skeletal muscle, super enhancers coordinate the differentiation direction of muscle fiber types by mediating the reprogramming of chromatin three-dimensional conformation and determining gene expression by regulating muscle fiber types such as MYH4 [128]. Beyond myofiber type regulation, research involving three-dimensional chromatin structure and SEs has rapidly expanded into other core areas of meat quality investigation, including intramuscular fat deposition, metabolism of meat flavor precursors, and regulation of postmortem muscle biochemical processes, further refining the molecular regulatory network governing meat quality traits. For instance, in studies related to high-grade marbling in beef cattle, researchers discovered that breed-specific SEs can mediate long-range interactions via chromatin loops to target the spatiotemporal expression of key adipogenic genes such as PPARG and FABP4, thereby determining the efficiency of intramuscular fat deposition and marbling scores [129]. In elucidating the meat quality of indigenous Chinese livestock and poultry breeds, research has confirmed that differences in three-dimensional chromatin structure among breeds constitute a crucial epigenetic basis for the unique meat flavor characteristics of local breeds. For example, a comparative study on skeletal muscle from Meishan pigs and Large White pigs revealed that approximately 75% of enhancers bypass the linearly closest gene and, through chromatin loops, remotely regulate more distant meat quality-related target genes. This finding fundamentally overturns the “nearest gene” functional inference principle commonly used in traditional GWAS studies, providing an essential tool for functional annotation of non-coding genetic variants [130]. At the same time, the study revealed the key role of the STARD7 gene in driving the transformation of glycolytic muscle fibers into oxidative ones, which provides a new epigenetic perspective for understanding meat quality differences [128]. Table 3 summarizes the core functional genes influencing meat quality traits. In summary, through the integration of multi-omics data, the existing research has systematically revealed the molecular basis of the formation of meat quality traits in various animals. The in-depth exploration from key genes and genetic variation to the three-dimensional genome level not only deepens our understanding of the genetic nature of variety differences but also provides a rich theoretical basis and technical support for the formulation of molecular marker-assisted breeding and precision breeding strategies.

## 6. Strategies for Meat Quality Improvement

The strategy of meat quality improvement is changing from traditional methods to the precise regulation of multi-omics guidance. By integrating transcriptome, metabolome and other data, the molecular network of meat quality traits is systematically analyzed, which provides new ideas for genetic breeding, nutritional intervention and environmental management.

### 6.1. Nutritional Regulation Strategies

Precise nutrition intervention can target the regulation of muscle metabolism after slaughter and improve meat quality. Vitamin E inhibits PSE meat formation by maintaining cell membrane integrity, and its effects of reducing drip loss and alleviating heat stress have been confirmed in pigs, poultry and other species [138]. As a calcium antagonist, magnesium reduces the incidence of PSE meat by regulating the glycolysis rate without affecting production performance [139]. As a precursor of serotonin, tryptophan supplementation can reduce stress response and significantly reduce the proportion of PSE carcasses [140]. In addition, the regulation of dietary composition affects muscle fiber type and glycogen metabolism, providing a basis for the formulation of multidimensional nutrition strategies. Furthermore, there are also certain limitations. The intervention effects exhibit strong context dependency, and the efficacy of additives is significantly influenced by factors such as breed, growth stage, and rearing environment, making it difficult to establish universally applicable standardized protocols. Moreover, most interventions can only mitigate meat quality deterioration, offering limited improvement in premium core meat quality traits such as intramuscular fat content and flavor compound composition.

### 6.2. Gene Marker-Assisted Selection

The application of molecular markers has greatly improved breeding efficiency. Important markers include those with expression levels positively correlated with average daily gain but negatively correlated with meat quality: the *PPP1R11* gene associated with meat quality, and polymorphisms in the Osteocrin gene linked to meat quality traits in Qinchuan cattle. Multi-omics integrated analysis further elucidates gene–phenotype regulatory networks, facilitating the construction of predictive models for meat quality. Table 4 summarizes core gene markers for meat quality improvement. Metabolomic markers can more directly reflect physiological states, and their correlations with parameters such as pH and intramuscular fat content provide a new dimension for selection. It must be emphasized that markers require functional validation and stability assessment to ensure their reliability across populations and environments. However, the effects of most functional genes and molecular markers show strong population specificity, with their applicability across different breeds or hybrid lines diminishing significantly or even disappearing entirely. The application of genomic selection is costly, requiring large reference populations and extensive multi-omics sequencing data, making it difficult for small- and medium-sized breeding enterprises to afford it. Currently, selection efforts remain primarily focused on genomic markers, while the transgenerational stability of epigenetic markers has not been fully validated, presenting technical bottlenecks for industrial application.

### 6.3. Environmental Optimization Protocols

Feeding system and density significantly affected meat quality. Studies have shown that the ground feeding density of more than 4/m^2^ leads to weight loss and increased pH of breast meat. Cage stocking density must be optimized to balance growth performance and meat quality. Feeding density changes water retention and tenderness by affecting glycogen storage and stress levels. Water quality management is also critical. Open waters contribute to weight gain, while closed systems need to balance health and animal welfare. Environmental stress can also change the level of protein phosphorylation, which in turn affects the tenderness and water-holding capacity of meat. Therefore, reducing stress is the core goal of environmental optimization [7]. However, the optimization of welfare-oriented environments significantly increases production costs and reduces unit breeding efficiency, limiting its promotion in large-scale commercial livestock operations due to economic constraints. Furthermore, the impact of environmental factors on meat quality is modulated by genetic background, resulting in markedly different outcomes for the same intervention across various breeds, which poses a challenge in formulating unified and standardized protocols.

## 7. Research Perspectives

Meat quality research is transitioning into a stage of precision and translational application, focusing on bridging the gap between molecular mechanism discovery and industrial breeding application, as well as establishing standardized evaluation systems for meat quality traits. However, it still faces challenges such as incomplete coverage of metabolomic detection, a lack of data integration standards, and inadequate cross-species analysis frameworks. In the future, it is necessary to focus on the development of high-sensitivity mass spectrometry technology and new computing platforms, and promote the transformation of the research paradigm from hypothesis-driven to data-driven with the help of machine learning.

### 7.1. Technological Development Trends

The technological development of meat quality research is undergoing profound changes from macro to micro, from single-omics to multi-dimensional integration. Single-cell transcriptomics is the first to reveal the cellular heterogeneity of muscle tissue, while spatial transcriptome and spatial metabolome technologies further locate gene expression and metabolite distribution to specific anatomical regions, intuitively explaining the spatial basis of intramuscular fat deposition and flavor substance formation. Proteomics, especially spatial proteomics technology, has become a key bridge between gene instructions and final phenotypic functions. The core of the future trend is to integrate single-cell/spatial multi-omics data to construct a “gene–protein–metabolite–space” four-dimensional regulatory network. This process is highly dependent on the continuous advancement of high-resolution detection technology and artificial intelligence-driven multimodal data integration algorithms, and this integration ultimately enables the accurate analysis and regulation of meat quality traits from a cellular mechanism perspective.

### 7.2. Unresolved Scientific Questions

A central challenge is extracting biologically meaningful mechanistic insights from large-scale datasets. A large number of unknown metabolites (especially flavor and lipid components) in metabolomics are difficult to identify due to the lack of standard maps. The construction of a gene regulatory network is limited by tissue-level data, and it is difficult to reflect cell heterogeneity. Species specificity (such as the unique response of Peking Duck to blue light) requires independent research and cannot be simply applied to the model. The existing data integration methods are mostly suitable for binary phenotypes, and the ability to deal with complex continuous traits, such as meat quality, is insufficient. In the future, it is necessary to break through the construction of a single-cell resolution network towards the development of livestock- and poultry-specific metabolite databases and analysis methods suitable for continuous phenotypes.

### 7.3. Predicting Future Core Research Directions

The core objective of future multi-omics research on meat quality is to overcome the critical bottleneck of extracting biologically meaningful mechanistic information from massive datasets. This endeavor will focus on verifiable research in three main directions: First, to address the challenge of identifying unknown metabolites related to flavor and lipids due to the lack of standard reference spectra, the approach will involve constructing a dedicated standard spectral library for livestock and poultry meat quality metabolites using a combination of GC-MS/LC-MS multi-platform non-targeted metabolomics and standard compound validation. Second, to overcome the limitations of tissue-level data, which fail to capture cellular heterogeneity and result in insufficient precision in constructing gene regulatory networks, the strategy would be to integrate single-cell transcriptomics with spatial multi-omics technologies. This will enable the construction of meat quality regulatory networks at single-cell resolution and elucidate the intrinsic mechanisms underlying the interactions between muscle cell subpopulations. Third, to address the inadequacy of existing data integration methods, which are mostly tailored for binary phenotypes and have limited capacity for handling complex continuous traits like meat quality, coupled with the bias from applying species-specific models indiscriminately, the plan is to develop multimodal data integration algorithms suitable for continuous meat quality traits while accounting for species specificity, based on graph neural networks. This will establish a standardized multi-omics analysis system applicable across different species.

### 7.4. Application Prospects Analysis

Multi-omics integration brings revolutionary potential to animal husbandry. In breeding, the causal network based on phenotypic, genotypic and transcriptome data can accurately analyze the mechanisms of fat deposition and muscle development and guide variety breeding. In feeding management, by identifying key metabolic markers and regulatory genes, a quantitative relationship model between environmental parameters (such as light and temperature) and meat quality can be established to optimize breeding strategies. In terms of quality evaluation, the integration of molecular markers and metabolic characteristics can construct an objective prediction model to achieve accurate classification based on the molecular level. With the application of single-cell sequencing and spatial transcriptome technology, researchers will be able to explore the effects of muscle tissue heterogeneity and local microenvironment on meat quality at a higher resolution level, providing more accurate guidance for animal husbandry production practice.

## Figures and Tables

**Table 1:** Measurements of key indicators of change in postmortem beef quality.

Indicator	Core Assay Method	Post-Mortem Change Trend	Relationship with Other Indicators	Quality Impact
pH Value [14]	Dynamic monitoring with insertion of pH meter	Decreases initially, then increases, with a minimum at day 4 (5.37 ± 0.03)	Correlated with decreasing ATP, AMP, and NADH levels	Determines muscle protein denaturation degree and water-holding capacity (WHC)
Moisture Content [16]	Direct drying method (national standard)	Slowly decreased during post-mortem aging, ranging from 72% to 76%	Negatively correlates with pH change and MFI	Determines juiciness and economic value
WBSF [14,22]	AMSA standard method, sample core heated to 71 °C	Increases then decreases, peaks at day 4 (157.94 ± 2.53 N)	Significantly negatively correlates with MFI	Core objective indicator of beef tenderness; <4.3 kg defined as “tender” by industry standard
MFI [14]	Proteomic analysis	Continuously increased during post-mortem aging, opposite to the trend of WBSF	Associated with CTSD, PSMD13, and 5 other proteins	Characterizes myofibril degradation degree, predicts tenderness and WHC
Energy Metabolites (ATP/AMP) [15]	^31^P nuclear magnetic resonance	Significant decrease within 0–2 days postmortem, maintained at low level thereafter	Drives pH decline and IMP accumulation	Influences muscle tenderization process
Protein Degradation Marker (SDS-PAGE)	Gel electrophoresis + Western blotting	Abundance of 30 kDa troponin T characteristic band increased significantly with aging time	Negatively correlated with WBSF, extremely significantly positively correlated with MFI	Core biomarker for post-mortem tenderization process

**Table 2:** Summary table of key information on core metabolic pathways for meat quality.

Core Metabolic Pathway	Key Regulatory Genes	Core Metabolic Markers	Associated Core Meat Quality Traits
Glycolysis and Energy Metabolism [16]	*HK2*, *PFKM*, *LDHA*	Glycogen, Lactate, ATP, Pyruvate	Postmortem pH value, Water-holding capacity, Incidence of PSE/DFD meat
Amino Acid Metabolism [31]	*GDH1*, *AST*, *BCAT2*	Glutamate, Aspartate, Branched-chain amino acids, Taurine	Umami flavor, Tenderness, Antioxidant capacity, Nutritional quality
Lipid Metabolism [55]	*SCD1*, *FADS2*, *FAS*, *ACC*	Triglycerides, Unsaturated fatty acids, Cholesterol	Intramuscular fat content, Marbling, Flavor, Nutritional value
Nucleotide Metabolism [27,28]	*AMPD1*, *ADSL*	IMP, ATP, Inosine, Hypoxanthine	Umami taste characteristics, Postmortem tenderization process
Redox Metabolism [89]	*Nrf2*, *GSH-Px*, *SOD1*	Glutathione, Malondialdehyde (MDA), ROS	Meat color stability, Storage performance, Tenderness, Flavor stability

**Table 3:** Core functional genes affecting meat quality.

Gene Name	Functional Category	Key Associated Meat Quality Traits	Core Mechanism of Action
*RYR1* [131]	Glycolysis/Abnormal Meat Regulation	PSE meat incidence, post-mortem pH, water-holding capacity (WHC)	Encodes sarcoplasmic reticulum calcium release channel; gain-of-function mutation accelerates glycolysis under stress
*PRKAG3* [131]	Glycolysis/Abnormal Meat Regulation	Muscle glycogen content, post-mortem pH, and acid meat incidence	Encodes AMPK γ3 regulatory subunit; dominant mutation causes abnormal glycogen accumulation in skeletal muscle
*CAST* [132]	Tenderness Regulation	Tenderness (shear force), WHC	Encodes endogenous calpain inhibitor; suppresses μ-calpain activity, high expression reduces meat tenderness
*CAPN1* [132]	Tenderness Regulation	Tenderness (shear force), post-mortem aging rate	Encodes core post-mortem tenderization enzyme; degrades myofibrillar structural proteins to drive meat tenderization
FABP3 [129]	Lipid Metabolism/IMF Regulation	IMF content, marbling score	Encodes fatty acid-binding protein; regulates fatty acid transport and lipid deposition in skeletal muscle
*FABP4* [129]	Lipid Metabolism/IMF Regulation	IMF content, marbling score	Specifically expressed in adipocytes; regulates adipocyte differentiation and fatty acid storage
*MSTN* [40]	Myofiber Development/Muscle Growth Regulation	Growth rate, dressing percentage, tenderness	Core negative regulator of skeletal muscle growth; loss-of-function mutation causes double-muscling phenotype and improves tenderness
*FASN* [133]	Lipid Metabolism/IMF Regulation	IMF content, fatty acid profile	Encodes rate-limiting enzyme for de novo fatty acid synthesis
*SCD* [86]	Lipid Metabolism/IMF Regulation	Fatty acid profile, meat flavor	Encodes rate-limiting enzyme for monounsaturated fatty acid synthesis
*MYH4* [128]	Myofiber Development/Muscle Growth Regulation	Myofiber type, tenderness, WHC	Encodes myosin heavy chain of glycolytic fast-twitch myofibers; high expression impairs meat quality
*MYH7* [128]	Myofiber Development/Muscle Growth Regulation	Myofiber type, tenderness, flavor quality	Encodes myosin heavy chain of oxidative slow-twitch myofibers; high expression improves core meat quality traits
*AMPD1* [134]	Umami and Flavor Regulation	Inosine monophosphate (IMP) content, meat umami	Encodes rate-limiting enzyme for IMP synthesis; determines muscle IMP accumulation
*ADSL* [135]	Umami and Flavor Regulation	IMP content, meat umami	Participates in purine nucleotide de novo and salvage synthesis; affects muscle IMP accumulation
*MC1R* [136]	Meat Color/Oxidative Stability Regulation	Meat redness, color stability	Encodes melanocortin 1 receptor; regulates melanin synthesis, significantly associated with muscle myoglobin content and meat redness
*NFE2L2 (Nrf2)* [137]	Meat Color/Oxidative Stability Regulation	Oxidative stability, shelf life	Core transcription factor of cellular antioxidant response; activates antioxidant enzyme expression to scavenge reactive oxygen species (ROS)
*PPP1R11* [16]	Glycolysis/Growth-Meat Quality Coregulation	Growth rate, post-mortem pH, tenderness	Regulates myofiber development and post-mortem glycolysis; balances growth performance and meat quality
*STARD7* [128]	Myofiber Development/Muscle Growth Regulation	Myofiber type, tenderness, WHC	Mediates mitochondrial lipid metabolism reprogramming; drives glycolytic-to-oxidative myofiber conversion

**Table 4:** Core gene markers for meat quality improvement.

Gene Name	Marker Type	Associated Core Meat Quality Traits
*PPP1R11* [16]	Transcript expression marker, SNP	Postmortem pH value, Drip loss, Tenderness, Intramuscular fat content
*CAPN1* [132]	SNP marker, InDel marker	Tenderness, Postmortem myofibrillar degradation rate
*CAST* [132]	SNP marker, Transcript expression marker	Tenderness, Water-holding capacity, Incidence of PSE meat
*MSTN* (*Myostatin*) [40]	InDel marker, SNP marker	Muscle fiber diameter and density, Tenderness, Intramuscular fat content, Slaughter rate
*MYH4* [128]	Transcript expression marker, SNP marker	Muscle fiber type composition, Tenderness, Water-holding capacity, Postmortem pH stability
*STARD7* [128]	Transcript expression marker, SNP marker	Muscle fiber type transformation, Tenderness, Intramuscular fat content
*FASN* [133]	SNP marker	Intramuscular fat content, Fatty acid composition, Meat flavor
*FABP4* [129]	SNP marker, Transcript expression marker	Intramuscular fat content, Marbling score
*AMPD1* [134]	SNP marker	Meat umami taste (inosine monophosphate (IMP) content), Postmortem glycolysis, pH stability
*IGF2* [141]	Imprinted gene/QTL	Muscle growth, Carcass lean meat percentage
*SELENBP1* [142]	Promoter region SNPs	Intramuscular fat content, Drip loss, Water-holding capacity

## Data Availability

No new data were created or analyzed in this study. Data sharing is not applicable to this article.

## Abbreviations

The following abbreviations are used in this manuscript:

CA	Component Analysis
CTSD	Cathepsin D
FMT	Fecal microbiota transplantation
GC-MS	Gas Chromatography–Mass Spectrometry
HSP	Heat shock protein
IGF-1	Insulin-like growth factor 1
IMF	Intramuscular fat
LC-MS	Liquid Chromatography–Tandem Mass Spectrometry
MFI	Myofibrillar Fragmentation Index
NMR	Nuclear magnetic resonance
PCA	Principal Component Analysis
RNA-Seq	Ribonucleic Acid Sequencing
ROS	Reactive oxygen species
SE	Super-Enhancer
SNP	Single-nucleotide polymorphism
SMS	Single-Molecule Sequencing
SSF	Slice shear force
TGF-β	Transforming growth factor β
WBSF	Warner–Bratzler force
